# Forensic Age Estimation through a DNA Methylation-Based Age Prediction Model in the Italian Population: A Pilot Study

**DOI:** 10.3390/ijms24065381

**Published:** 2023-03-11

**Authors:** Martina Onofri, Arianna Delicati, Beatrice Marcante, Luigi Carlini, Federica Alessandrini, Pamela Tozzo, Eugenia Carnevali

**Affiliations:** 1 Section of Legal Medicine, Department of Medicine and Surgery, Santa. Maria Hospital, University of Perugia, 05100 Terni, Italy; 2 Department of Cardiac, Thoracic, Vascular Sciences and Public Health, University of Padova, 35122 Padova, Italy; 3 Department of Biomedical Sciences and Public Health, Polytechnic University of Marche, 60121 Ancona, Italy

**Keywords:** DNA methylation, SNaPshot, age estimation, forensic genetics, epigenetics

## Abstract

DNA methylation is one of the epigenetic marks which has been studied intensively in recent years for age predicting purposes in the forensic area. In order to integrate age prediction into routine forensic workflow, the purpose of this study was to standardize and optimize a DNA methylation-based protocol tailored to the Italian context. A previously published protocol and age-predictive method was implemented for the analysis of 84 blood samples originating from Central Italy. The study here presented is based on the Single Base Extension method, considering five genes: ELOVL2, FHL2, KLF14, C1orf132, now identified as MIR29B2C, and TRIM59. The precise and specific steps consist of DNA extraction and quantification, bisulfite conversion, amplification of converted DNA, first purification, single base extension, second purification, capillary electrophoresis, and analysis of the results to train and test the tool. The prediction error obtained, expressed as mean absolute deviation, showed a value of 3.12 years in the training set and 3.01 years in the test set. Given that population-based differences in DNA methylation patterns have been previously reported in the literature, it would be useful to further improve the study implementing additional samples representative of the entire Italian population.

## 1. Introduction

In the last ten years, forensic genetics techniques, that enable investigators to infer additional traits from an unidentified specimen, have been developed to reduce the number of suspects, generate fresh leads in cold cases, or identify an unknown person or mass casualty victims. In particular, these approaches concentrate on age, phenotypic traits (such as eye, skin, and hair color), and biogeographic origin prediction. While the phenotypic characteristics and biogeographical origins are mainly studied through the analysis of specific SNP groups, age prediction is primarily conducted through the analysis of specific epigenetics patterns [1,2].

DNA methylation is one of the epigenetic marks and it has been studied intensively in recent years; in particular, measuring the DNA methylation levels of various genes is very important across many forensic and medical areas. As already mentioned, the survival, growth, and differentiation of cells are regulated by changes in the epigenetic state of specific genes [3].

Assuming that scientific and technological progress in human epigenomics continues to accelerate, we can imagine the establishment of an “epigenomic fingerprint” from crime scene traces to answer various forensically relevant questions that cannot be detected only through genetics. Furthermore, we expect to involve predicting forensically valuable lifestyle and environmental information of an unknown trace donor in the near future [4].

Nowadays, the three main forensic applications of methylation analysis are tissue type determination of human biological traces, differentiation of monozygotic twins, and age prediction of an unknown donor [5].

Aging is a very complex phenomenon; it is multi-dimensional in nature, from a qualitative, quantitative, and inter-individual point of view [6]. We now have evidence that aging is, at least in part, genetically and epi-genetically controlled. The control and subsequent interruption of cellular activities demonstrate the cell’s programmed decision to continue or discontinue maintenance procedures as it ages [7]. A chronological age clock counts the years since birth. Individuals of the same chronological age may have distinct biological ages and might have quite different amounts of age-related dysfunction, pathology, and mortality risk and be considered to be of varied biological age. The scale of chronological or biological age may match or diverge depending on both the individual’s lifestyle and the presence of disease. While chronological age has been shown to be a valuable tool in forensics, biological age may also be used to track the development of a person suffering from an illness or seeking therapy for a medical condition [8].

Initially, clock models were created using a single sort of biomarker, that is DNA methylation, which was used to predict chronological age. This first generation is referred to as “chronological clocks”. A second generation of epigenetic clocks has been developed that utilizes DNA methylation to forecast biological qualities such as time to death or functional deterioration. These so-called “biological clocks” have been demonstrated to predict better outcomes in specific disease patterns [8,9,10].

**Figure 1:**
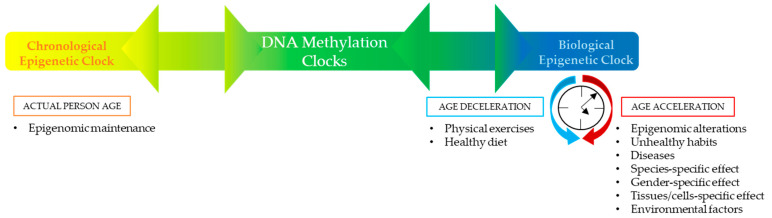
Modulation of DNA Methylation Clocks.

Different factors may contribute to defining individuals’ DNA methylation clocks. The chronological component is associated with epigenomic maintenance, which guarantees a most precise forensic age determination. Meanwhile, the biological component may be positively influenced by physical exercises and a healthy diet, or negatively influenced by epigenomic alterations, unhealthy habits (smoking, alcohol consumption, sedentariness), diseases, species-specific effect, gender-specific effect, tissues/cells specific effect, and environmental factors (air pollution, temperature, humidity, UV exposure, and pathogens). Therefore, separating aging into distinct chronological and biological components has been challenging, such that all clocks lay between the two extremes. Relative to this concept, as illustrated in the figure below (Figure 1), defining the chronological and biological drivers of these DNA methylation clocks will need a deep analysis separately. The precise separation of these two elements due to particular sets of CpGs would result in more powerful specialized clocks and independent mechanistic research, especially in the case of legal measures of the human age [4,9,10,11,12,13].

Another important concept in the study of epigenetics includes the distinction between epigenetic drift and the epigenetic clock. In fundamentally separate mechanisms, epigenetic drift and the epigenetic clock contribute to age-related DNA methylation alterations [14,15].

The study here presented is based on the Single Base Extension method, considering five genes: ELOVL2 (located on 6p24.2), FHL2 (located on 2q12.2), KLF14 (on chromosome 7q32), C1orf132, now identified as MIR29B2C (located on 1q32.2), and TRIM59 (located on 3q25.33).

This study aimed to predict the age of different individuals starting from human biological samples collected from Italian volunteers. The development of this analysis method was based on the original DNA methylation works of Jung et al. [16] and Cho et al. [17]. However, some modifications to the primer mixes for both the PCR and SBE reactions, and to the annealing temperature were performed in order to optimize the protocol for our laboratory. As stated above, the DNA methylation clock is influenced by different factors; therefore, the age-predictive models previously proposed in the literature need to be shaped on the specific population [16,17]. For this reason, a literature study was considered necessary for the creation of a DNA methylation analysis protocol for forensic application in the Italian context, where a specific protocol in this field has not yet been proposed nor validated.

## 2. Results

**Figure 2:**
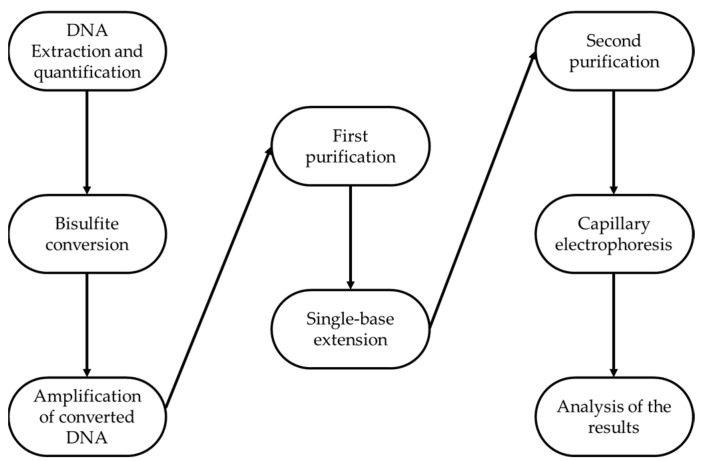
Experimental workflow.

A pool of 84 peripheral blood samples was analyzed in replicates following precise and specific steps consisting on DNA extraction and quantification, bisulfite conversion, amplification of converted DNA, first purification, Single Base Extension, second purification, capillary electrophoresis, and lastly, analysis of the results (as reported in Figure 2).

A DNA extract of each sample was bisulfite-converted twice and each converted eluate was amplified twice, for a total of four replicates per individual sample. The four amplification products followed the same downstream process and the SBE step was repeated once for each of them. After capillary electrophoresis, the methylation levels at each locus (namely, ELOVL2, FHL2, KLF14, C1orf132/MIR29B2C, TRIM59) were calculated and initially inserted in the tool developed by Lee et al. [18]. However, given the modifications performed on the original protocol and the different populations studied, the data thus obtained were used to create a novel age-predictive tool. The samples’ replicates were considered independent data, bringing the total number to 336 observations. Two-thirds of the 336 samples were used as a training set to create a multivariate linear regression model, and the remaining one-third of the samples made up the test set. Replicates of the same sample were included in the same dataset.

**Figure 3:**
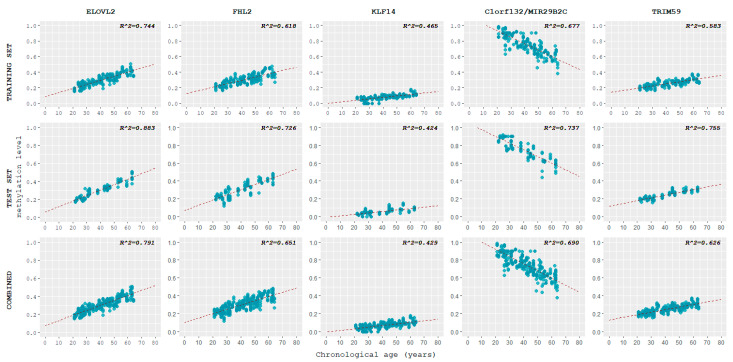
Scatterplots displaying the correlation of DNA methylation levels at each of the five loci studied with chronological age for all three datasets.

For the training set, methylation levels vs. chronological age were plotted per each locus using the ggplot2 package in R (Figure 3).

Pearson correlation coefficients r between chronological age and methylation levels at each locus were calculated and they were evaluated according to the same rule of thumb adopted by Jung et al. [16] (Table 1). Four loci, ELOVL2, C1orf132, FHL2, and TRIM59, showed a strong correlation between chronological age and methylation levels, while KLF14 showed a moderate correlation.

As a reference, the correlation between chronological age and methylation patterns in the test set and in the combined dataset (training and test set samples) was plotted (Figure 3) and calculated (Table 1). The values obtained for the test set were higher than those of the training set, especially in the case of TRIM59. An exception was KLF14, whose value was slightly lower. The combined dataset showed a correlation between DNA methylation levels and chronological age that mirrored the one observed in the training set.

A multivariate linear regression model was created with the training set using Microsoft Excel. The model was then applied to the test set for its validation. The multivariate predictive model allowed for an estimation of the age (predicted age) of individuals based on the methylation pattern of each gene, according to the following formula:

Predicted age (in years) = 73.8686066416537 × ELOVL2 DNA methylation level + 21.6625107564553 × FHL2 DNA methylation level + 47.5538289868233 × KLF14 DNA methylation level −37.6472633609841 × C1orf132/MIR29B2C DNA methylation level + 4.49257531145724 × TRIM59 DNA methylation level +35.8493598840537.

**Figure 4:**
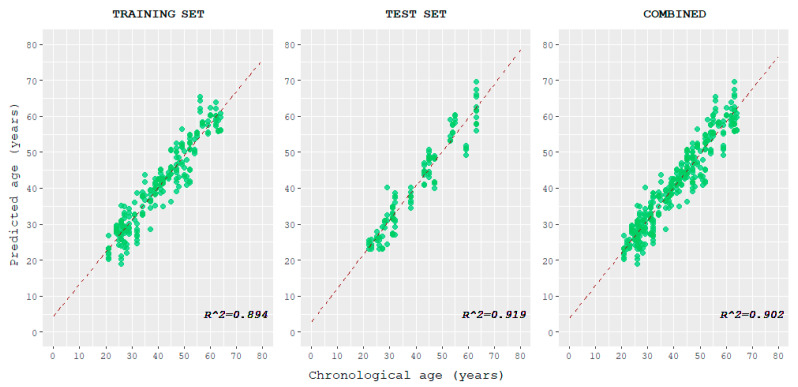
Scatterplots displaying the correlation between the age predicted using the tool here described and chronological age, for training, test, and combined datasets.

The correlation between predicted age and chronological age was plotted for the training, test, and combined datasets and evaluated (Figure 4 and Table 1). The correlations observed in the training set and test set resulted in strong and very strong values, respectively.

The prediction accuracy of the model was assessed for both the training set and test set separately, and for the combined set, by calculating the Mean Absolute Deviation (MAD) for all the samples pooled together and for each age category (Table 2). In the training set, the overall MAD had a value of 3.12 years, while the test set had a value of 3.01 years. As can be seen from the MAD value, the prediction model’s output was accurate and homogeneous, both for the training set and for the test set. With regard to MAD values of each age category, in the training set the lowest MAD of 2.79 years was found for the 41–50 age category while in the test set the lowest MAD of 2.41 years was found for the youngest category (18–30 years). The highest MAD of 3.67 and 4.09 years in training and test set, respectively, was observed for the 51–65 years of age category. The combined dataset showed the lowest MAD value in the 31–40 years of age category, while, as expected, the highest MAD was found in the 51–65 age category. The youngest category showed the third lowest error value.

The four replicates of each sample were then analyzed as a group, the MAD for each sample replicate group was calculated, and the results were evaluated based on the age category the samples belonged to (Table 2). The maximum value for the MAD was around 4 to 6 years, across all age categories; however, five individuals showed a MAD greater than 6 years, with a maximum value of around 8 years.

### Four Loci Model Construction

Given the strong correlation of TRIM59 with chronological age observed in the simple linear regression analysis, the results of the multivariate linear regression analysis highlighted a higher *p*-value for this locus (*p* = 0.374). Given this observation, a model with four CpG sites was built excluding TRIM59. The four loci model showed the same age correlation value as the model with five CpG sites (r = 0.945) explaining 89.2% of age variation (adjusted R^2^ = 0.892). Applying this model, the age prediction was obtained through the formula:

Predicted age (in years) = 74.816 × ELOVL2 DNA methylation level + 22.601 × FHL2 DNA methylation level + 49.334 × KLF14 DNA methylation level—37.6771 × C1orf132 DNA methylation level + 36.3226.

**Figure 5:**
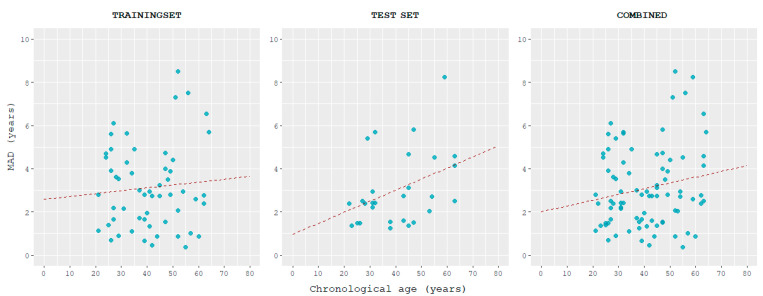
Correlation of MAD and chronological age in the training, test, and combined sets in the 4 markers model. Samples are sorted in ascending order of age.

This model was able to estimate age with a correlation between predicted and chronological ages of 0.945 and MAD of 3.13 years in the training set and 0.958 and 3.04 years in the test set. MAD values between predicted and chronological ages tended to increase with age, slightly in the training set, and more in the test set (Figure 5).

## 3. Discussion

Forensic genetics techniques’ recent advances have allowed investigators to acquire additional information from their samples to reduce the number of suspects, generate fresh leads in cold cases, or identify an unknown person or mass casualty victims. Among these innovative techniques, age estimation through DNA methylation has recently attracted great attention in the forensic community. The purpose of this study was to evaluate the individual epigenetic age clock at the level of specific CpG sites to create a predictive tool dependent on individual chronological age. Through a careful literature analysis, five relevant loci (ELOVL2, FHL2, KLF14, C1orf132, and TRIM59) were identified. At these loci, the methylation status of each individual was studied, and the protocol thus developed was used to create the prediction model for age estimation specifically in the Italian population. The samples were analyzed as replicates and as mentioned before, two-thirds were used for the training set, and thus for creating a multivariate linear regression model, whereas the remaining one-third was used as a test set to validate the tool.

In the training set samples, the DNA methylation levels at each locus were correlated with chronological age. Considering the linear regression trend of each of the five genes, it can be observed that all genes presented hypermethylation with increasing age, except for C1orf132, which showed a tendency to hypomethylation. This observation is in accordance with a previous study by Correia Dias et al., who reported a positive correlation with age for ELOVL2 and FHL2 genes, and a negative correlation with age for the C1orf132 gene [19]. The result of this observation was that, at C1orf132, younger people presented a much higher level of methylation which then progressively decreased with increasing age, while for the other four genes (ELOVL2, FHL2, KLF14, and TRIM59) the opposite was true: the percentage of methylation increased with age [3,20].

As shown in Table 1, a strong correlation for ELOVL2, FHL2, C1orf132, and TRIM59 genes (0.7 < |r| ≤ 0.9) was observed, whereas a moderate correlation resulted for the KLF14 gene (0.5 < |r| ≤ 0.7). These data are in agreement with what emerged from the study of Cho et al., where the Pearson correlation index in KLF14 was lower in comparison to the other genes analyzed [17].

The fact that the ELOVL2 gene had the highest Pearson coefficient reflects what has already been indicated in the literature: ELOVL2 has been heralded as one of the most reliable genes to be used in this type of analysis [17,21,22,23]. Indeed, Aliferi et al., highlighted the success of CpG markers located in the ELOVL2 gene region and how this is probably due to their larger methylation range which improves age prediction accuracy [24]. Moreover, other scholars have suggested its reliability as a multi-tissue age-predictive marker also [16,25,26].

The second strongest correlation was observed in C1orf132, for which the Pearson coefficient resulted to be –0.823. Comparing our results with those of Jung et al., it is possible to highlight a difference in the outcome. Indeed, our value fell within the strong range of correlation (0.7 < |r| ≤ 0.9) whereas Jung et al. had a moderate value (0.5 < |r| ≤ 0.7) [16]. This difference may be attributed to the different populations considered in the studies, suggesting for this locus a higher correlation between methylation status and age for the Italian population rather than the Korean one.

Additionally, a strong correlation for the FHL2 CpG site with individual chronological age was observed, suggesting its reliability for use in the age prediction tool. This was also highlighted by numerous studies which included the gene for the generation of an age-estimating model [16,17,27,28].

As for the TRIM59 gene, a strong correlation with chronological age was found in our population sample. This outcome is also supported by what was obtained by Jung and colleagues in a pool of volunteers of Korean nationality and in the study conducted by Zbiec-Piekarska on Polish people, albeit the latter analyzed a different CpG site [16,27].

Lastly, KLF14 gene methylation status had the second lowest Pearson correlation coefficient, with a value that fell within a moderate range of correlation (0.5 < |r| ≤ 0.7). Nevertheless, its correlation was approximately near to what was reported in the literature [16,17,27].

Our results were then compared with previously published data. Comparison of DNA methylation levels obtained in blood samples from Italian, Portuguese [29], and Korean individuals [16] showed a strong and very strong correlation (|r| > 0.7) with age for all the CpG sites, except for KLF14 in Italians and C1orf132 in Koreans (Table 3). The highest correlation value with age was obtained for ELOVL2 in Italians and Portuguese individuals and for FHL2 in Koreans.

In the multivariate analysis, the CpGs in KLF14 and TRIM59 genes showed non-significant age correlation values in Portuguese individuals; in Italians, only TRIM59 showed a non-significant correlation, while in Koreans all the five markers showed significant age correlation. Age correlation similarities (i.e., ELOVL2 in Italian and Portuguese individuals) and differences in specific markers suggest that methylation levels might be population-specific and specific loci may be more suitable for different population groups in order to estimate the chronological age.

Moreover, the predicted output of the model for each observation was correlated with the sample’s respective chronological age. A strong correlation between the two was observed in the training set, while a very strong correlation was observed in the test and combined sets. This suggests that the trend of the multivariate linear regression model, which allows age estimation, is actually influenced by the chronological age of individuals.

The prediction accuracy of the multivariate linear regression model, calculated as a MAD, was evaluated for both the training and test datasets. This evaluation was performed for the four sample replicates, considering them both as a single data observation and as a group. The prediction error obtained had a value of 3.12 years in the training set and 3.01 years in the test set. In addition, these values were slightly lower than those reported in the literature where the MAD values fell in the range from 3.48 to 5.75 years [16,22,27]. When considering the samples according to their age groups, an increase in the error range corresponding to an increase in the chronological age of the samples was observed. The progressively higher MAD value may be explained by the higher variability in methylation levels of older people due to the accumulation of environmental factors, stressors, and pro-methylation lifestyle habits.

The highest MAD observed per sample replicates was around 8 years for both the training set and test set. Five were the samples with a Mean Absolute Deviation greater than 6 years. These results were cross-referenced with the questionnaires filled out by volunteers. It was noted that, in 80% of the cases (4 out of 5 samples), the high MAD values observed may be attributed to past or present smoking habits. Indeed, as already mentioned, many scholars have highlighted how these habits may influence the epigenetic modifications of individuals [4,10,11,12]. However, other factors, which are not currently under investigation, may be additional causes, which may have led to these results given the high complexity of epigenetic regulation.

Indeed, given the relevance of age prediction in the scientific community, different studies have focused on the development of age-predictive tools based on different types of potential aging-related biomarkers. Nonetheless, the study of DNA methylation patterns seems to be the most accurate and sensitive in terms of chronological age prediction and life span evaluation [24,30]. Moreover, despite the impossibility of controlling all the possible factors which can impact epigenetic variations, it remains important to outline a reliable tool for age estimation to infer additional information from an unidentified specimen, especially in the forensic context. However, even in this case, further studies are needed to ensure that this tool reaches levels of accuracy and greater sensitivity in order to minimize the divergences between chronological and predicted ages.

In our opinion, our findings have provided valuable insights that may serve as a starting point. Most importantly, given the quantitative nature of the bisulfite-dependent method of DNA methylation levels estimation, the analysis in replicates of the samples minimized variability in the results, thus showing an improved outcome in the predictive accuracy of the model. Additionally, our study can be considered as an opportunity to delve deeper into the definition of the most informative loci for age estimation in the Italian population. However, a number of subsequent steps would be appropriate to overcome some aspects that could represent limits to this study. First, the implementation of a more diverse pool of individuals, in terms of lifestyle habits, disease status, and environmental factors that may influence DNA methylation patterns is advisable. In fact, the possible correlation between these factors and methylation profiles has not been thoroughly investigated, which could affect the prediction accuracy of this tool. Second, given that the study was limited to 84 individuals, the investigation of a wider population, representative of the Italian one, would be needed in order to increase the sensitivity of the predictive model for age estimation, through, for example, the structuring of a multicenter collaborative study. In addition, considering the limited amounts of DNA typically encountered in the forensic context, further studies of the amount of starting material are needed to improve the accuracy and reliability of age prediction for forensic analysis.

In conclusion, given the observed variability among different populations, new specific markers are needed to better explain the age-related DNA methylation variance in different population groups; however, the variation due to environmental effects and diseases will always play a confounding role [31].

## 4. Materials and Methods

### 4.1. Sample Collection

The study was approved by the Ethical Committee of Perugia University, Umbria, Italy. Written informed consent to sample collection and analysis was provided by all volunteers. Additionally, volunteers filled out a general questionnaire pertaining to their age, gender, lifestyle, and known pathologies (without specifying which ones), as they are factors that may influence age prediction. The information was collected to aid the results’ analysis in case of any observed inconsistencies. Otherwise, 84 individuals known to be healthy were chosen for the study. Samples were collected from 44 females and 40 males aged 18–65, evenly distributed among four age classes: 18–30 years, 31–40 years, 41–50 years, 51–65 years of age. All volunteers were located in Central and Central-Northern Italy.

The samples consisted of peripheral blood samples collected with EDTA and they were processed right after collection.

### 4.2. DNA Extraction and Bisulfite Conversion

Aliquotes of 200 μL of blood were extracted using QIAamp^®^ DNA Mini Kit (Qiagen, Hilden, Germany) and the DNA extracts were quantified using the Quantifiler^TM^ Trio DNA Quantification Kit (Applied Biosystems^®^, Foster City, CA, USA). Optimally, around 400 ng total of DNA were bisulfite-converted using EZ DNA Methylation-Direct^TM^ Kit (Zymo Research, Irvine, CA, USA). A lower DNA yield was observed. Even if the kit’s manual reported a yield of more than 80%, we found it to be slightly lower, so we assumed the DNA recovery to be 60%.

The 84 samples were bisulfite-converted twice and both converted DNA eluates were amplified twice, for a total of four replicates per sample.

### 4.3. Amplification of Converted DNA

The primers implemented for PCR amplification were those used by Jung et al. [16], and Cho et al. [17], with modifications to the primers’ concentration mixtures. The modified primers’ concentration for the PCR amplification is reported in Table 4.

Since the primer concentrations were adjusted, the annealing temperature was recalculated by using a dedicated tool by Thermo Fisher Scientific [32]. The best results were obtained with the samples amplified with our modified primer mixture at a melting temperature of 54 °C. According to these specifics, four replicates of the same sample were amplified, and the results obtained were consistent, with only negligible deviations observed.

For the PCR enrichment step, based on the converted DNA input suggested by Jung et al. [16], 10 ng of converted DNA was amplified. Samples were diluted accordingly based on the assumed yield of the bisulfite conversion. For a single sample, the amplification reaction was prepared as follows:

6.25 µL QIAGEN^®^ Multiplex PCR Master Mix 2× (Multiplex PCR Kit);
1.25 µL PCR PRIMER MIX 10×;
1 µL H_2_O;
4 µL bisulfite-treated DNA.

The amplification conditions consisted of an initial denaturation at 95 °C for 10 min, followed by 45 cycles at 95 °C for 30 s, 54 °C for 30 s, and 72 °C for 30 s, and lastly an extension at 72 °C for 5 min, then hold at 4 °C.

### 4.4. SNaPshot Protocol

The PCR amplification step was followed by an enzymatic cleanup, carried out by the ExoSAP-IT^TM^ Express PCR Product Cleanup reagent (Thermo Fisher Scientific, Waltham, MA, USA), according to the manufacturer’s instructions.

The multiplex SBE reaction was carried out using the SNaPshot^TM^ Multiplex Kit (Thermo Fisher Scientific, Waltham, MA, USA). The same primers of Jung et al. [16], and Cho et al. [17] were used, albeit their concentrations in the 10× primer mix were also modified. All 100× SBE primers were diluted to achieve 10× primers at a concentration of 0.2 μM. Based on the SNaPshot™ Multiplex Protocol [33], the SBE reaction per each sample wasprepared as follows:

5 µL SNaPshot™ Multiplex Ready Reaction Mix;
1 µL SBE PRIMER MIX 10×;
1 µL H_2_O;
3 µL ExoSAP™ purified DNA.

The temperature conditions in the thermal cycler for SBE were set as per the manufacturer’s instructions, and they consisted of 25 cycles of rapid temperature increase to 96 °C, hold at 96 °C for 10 s, rapid temperature decrease to 50 °C, hold at 50 °C for 5 s, rapid increase to 60 °C, hold 60 °C for 30 s, lastly a final rapid decrease to 4 °C and hold at 4 °C until post-SBE purification.

The SNaPshot multiplex reaction was followed by enzymatic purification with Shrimp Alkaline Phosphatase (SAP) (Thermo Fisher Scientific, Waltham, MA, USA) according to the manufacturer’s instructions, meaning an incubation at 37 °C for 30–60 min. The reaction mix per sample was the following:

12 µL H_2_O;
2 µL 10x SAP Reaction Buffer;
1 µL SAP;
5 µL Post-SBE Product.

### 4.5. Capillary Electrophoresis

Capillary electrophoresis was carried out on the SeqStudio^TM^ Genetic Analyzer, with a POP-1 universal polymer. The GeneScan E5 module parameters were the same as the kit’s protocol; however, the collection time was shortened from 24 min to 18 min. Firstly, post-SAP SBE products were prepared in 0.5 μL sample tubes according to the following mix for a single sample:

15 μL Hi-Di formamide;
0.15 μL GeneScan™-120 LIZ™ size standard;
1.5 μL post-SAP SBE product.

The sample data are then analysed by using GeneMapper^®^ Software v 6.

### 4.6. Age Calculation and Prediction Model Construction

DNA methylation-based age prediction depends on the methylation levels at particular CpG sites. The methylation degree at ELOV2 and FHL2 CpG sites, given that C and T are detected, was calculated according to the formula:

$$\frac{I_{C}}{I_{C}+I_{T}}.$$

At KLF14, C1orf132 and TRIM59 sites, which had their SBE primers designed in reverse and thus G and A were detected, the methylation levels were calculated using the formula:

$$\frac{I_{G}}{I_{G}+I_{A}}.$$

where I is the intensity of either the methylated C or unmethylated C, meaning the height of their electrophoretic peaks.

## 5. Conclusions

The main purpose of this study was to implement a protocol and method for age prediction, and to define an age-predictive tool for the Italian population, given that some differences among methylation levels in different populations were observed. For these reasons, after a precise literature analysis we implemented a study protocol optimal for our laboratory and trained and tested the tool with samples originating from Central Italy. The samples were analyzed as replicates to determinate the DNA methylation level highlighting a strong correlation both with chronological and predictive age, either in the training set or in the test set. The deviation between the predicted age and the chronological age was calculated through Mean Absolute Deviation given a mean value of approximatively 3 years suggesting the reliability of the predictive model. Moreover, based on our experience, to minimize the error rate of each sample, replicated analyses are suggested. Eventually, despite our purpose to standardize DNA methylation analyses, protocol, and interpretation patterns for forensic application in the Italian context, it would be useful to implement the study with additional samples collecting more information about donors that would be helpful for a future more detailed analysis.

## Figures and Tables

**Table 1:** Pearson correlation coefficient r of DNA methylation level variations with chronological age and predicted in each of the five loci studied, for the training, test, and combined datasets. Correlation for predicted age with chronological age in the three data sets is also displayed.

	Correlation of DNA Methylation Levels with Chronological Age					Correlation of Predicted Age with Chronological Age
	ELOVL2	FHL2	KLF14	C1orf132	TRIM59	
TRAINING SET	0.862	0.786	0.682	−0.823	0.753	0.779
TEST SET	0.940	0.852	0.651	−0.858	0.869	0.958
COMBINED	0.889	0.807	0.655	−0.831	0.791	0.950

**Table 2:** Mean Absolute Deviation (years) values calculated for both datasets separately in their entirety and after dividing the samples into age classes (top rows). MAD was also calculated considering the sample replicates as a group and according to their age class (bottom rows). The same analysis was applied to the combined data set.

Mean Absolute Deviation (MAD) in Years								
	Entire dataset	MAD per age class						
		18–30	31–40	41–50	51–65			
TRAINING SET	3.12	3.14	2.84	2.79	3.67			
TEST SET	3.01	2.41	2.57	2.97	4.09			
COMBINED	3.08	2.91	2.74	2.85	3.78			
	MAD per age class per sample replicates							
	18–30		31–40		41–50		51–65	
	MIN	MAX	MIN	MAX	MIN	MAX	MIN	MAX
TRAINING SET	0.67	6.10	0.70	5.81	0.46	4.77	0.35	8.53
TEST SET	1.31	5.33	1.22	5.50	1.34	5.84	2.03	8.24
COMBINED	0.67	6.10	0.70	5.81	0.46	5.84	0.35	8.53

**Table 3:** Comparison of age correlation values in blood samples from individuals of Italian, Portuguese, and Korean ancestry. For all populations, the analysis was performed at the same CpG sites.

Locus	Italians		Portuguese		Koreans	
	R	R^2^	R	R^2^	R	R^2^
ELOVL2	0.862	0.744	0.951	0.904	0.879	0.773
FHL2	0.786	0.618	0.946	0.895	0.893	0.797
KLF14	0.682	0.465	0.791	0.625	0.777	0.604
C1orf132	−0.823	0.677	−0.924	0.854	−0.637	0.406
TRIM59	0.753	0.567	0.910	0.828	0.763	0.582

**Table 4:** First amplification 10× primer mix forward and reverse primers’ concentration.

AMPLIFICATION MIX	10× Primer Mix Concentrations	
	Fwd	Rev
ELOVL2	1 µM	1 µM
FHL2	0.5 µM	0.5 µM
KLF14	0.5 µM	0.5 µM
C1orf132/MIR29B2C	1 µM	0.5 µM
TRIM59	0.5 µM	0.5 µM

## Data Availability

The data presented in this study are available on request from the corresponding author.
